# Impact and oxidation of single silver nanoparticles at electrode surfaces: one shot *versus* multiple events†
†Electronic supplementary information (ESI) available: Summary of previous studies; Ag NP characterization: TEM and DLS; event duration histogram; maximum current histogram. See DOI: 10.1039/c6sc04483b



**DOI:** 10.1039/c6sc04483b

**Published:** 2016-12-12

**Authors:** Jon Ustarroz, Minkyung Kang, Erin Bullions, Patrick R. Unwin

**Affiliations:** a Department of Chemistry , University of Warwick , Coventry , CV4 7AL , UK . Email: p.r.unwin@warwick.ac.uk; b Vrije Universiteit Brussel (VUB) , Research Group Electrochemical and Surface Engineering (SURF) , Pleinlaan 2 , 1050 Brussels , Belgium . Email: justarro@vub.ac.be

## Abstract

Single nanoparticle (NP) electrochemical impacts is a rapidly expanding field of fundamental electrochemistry, with applications from electrocatalysis to electroanalysis. These studies, which involve monitoring the electrochemical (usually current–time, *I*–*t*) response when a NP from solution impacts with a collector electrode, have the scope to provide considerable information on the properties of individual NPs. Taking the widely studied oxidative dissolution of individual silver nanoparticles (Ag NPs) as an important example, we present measurements with unprecedented noise (< 5 pA) and time resolution (time constant 100 μs) that are highly revealing of Ag NP dissolution dynamics. Whereas Ag NPs of diameter, *d* = 10 nm are mostly dissolved in a single event (on the timescale of the measurements), a wide variety of complex processes operate for NPs of larger diameter (*d* ≥ 20 nm). Detailed quantitative analysis of the *I*–*t* features, consumed charge, event duration and impact frequency leads to a major conclusion: Ag NPs undergo sequential partial stripping (oxidative dissolution) events, where a fraction of a NP is electrochemically oxidized, followed by the NP drifting away and back to the tunnelling region before the next partial stripping event. As a consequence, analysis of the charge consumed by single events (so-called “impact coulometry”) cannot be used as a general method to determine the size of colloidal NPs. However, a proper analysis of the *I*–*t* responses provides highly valuable information on the transient physicochemical interactions between NPs and polarized surfaces.

## Abstract 2

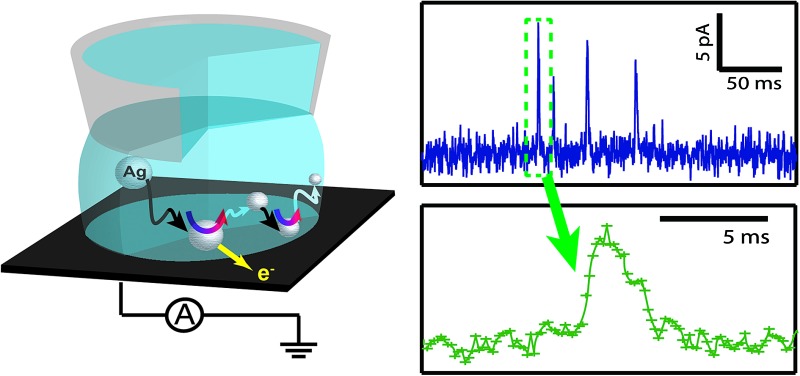
High bandwidth-low noise measurements of the electrochemical oxidation of individual silver nanoparticles (NPs) impacting on electrodes reveals the process to typically occur in a series of ‘bites’ (partial NP dissolution) rather than in a single shot, with the resulting current–time traces revealing considerable information on NP activity and transport near electrodes.

## 1 Introduction

Over the last decade, synthetic routes have been refined to the point where nanoparticles (NPs) can be produced with good control over size, shape and composition.^1,2^ Yet, developments in understanding the physicochemical and functional properties of NPs are still underway. This is particularly true for the electrochemistry of NPs, which is of fundamental interest and underpins a wide range of energy conversion and sensing devices.^3^ Gaining deeper knowledge of the relationship between size, structure and activity of NPs is essential for rational and optimised applications.

Traditionally, electrochemical characterization of NPs has involved measurements on large populations of NP ensembles immobilised on surfaces.^4^ However, the properties determined in this way are convoluted by subtle variations in NP size and shape, and potentially complex interactions between neighbouring NPs.^4^ As such, the electrochemical properties of single NPs and their interaction with different types of substrate electrodes is an important emerging area. A prominent method to study the electrochemistry of single NPs,^5^ relies on monitoring the electrochemical signal when an electroactive NP impacts from a suspension of NPs in solution onto a collector electrode surface, a rapidly growing area called single NP impact electrochemistry (SNIE). SNIE requires an experimental set up that has low background noise and low NP impact frequency, which has normally been achieved by the use of ultramicroelectrodes (UMEs) of a few microns in diameter.^6–9^

Hitherto, there are basically two approaches to monitor NP electrochemical impacts. First, electrocatalytic current amplification, which relies on an electrochemical process that is catalysed by the NP under study, but not the collector electrode (over the applied potential range of interest).^5,7–14^ The recorded current–time (*I*–*t*) transient can provide information on the interaction of the NP with the substrate (elastic collision, adsorption, *etc.*) as well as the kinetics of the reaction under study. If the kinetics are fast and the process is mass transport limited, the size of the colliding NP can also be inferred from the diffusion-limited current.^5,15^

Second is electrochemical oxidative dissolution (stripping).^16^ In this case, the measured current–time transient reflects the anodic dissolution of the NP into its constituent ions upon impact. By measuring the charge consumed during each NP collision, it has been proposed that the size of the colliding NP can be inferred by Faraday's law.^16–21^ However, this requires that the *I*–*t* transient is measured with high accuracy and that the whole particle is oxidized while in contact with the surface in a single impact.

Complete NP dissolution has been claimed for Ag particles with diameters ranging from 6 nm (ref. 20) to 100 nm,^18^ and it has consequently been argued that impact coulometry can be used as a method for *in situ* determination of the size, with nm resolution, of colloidal NPs of different materials such as Ag,^18–20^ Cu,^22^ Mo,^23^ C-60,^24^ Au^25^ or to address colloidal stability and aggregation phenomena.^17,21^ However, none of these studies provided a thorough analysis of the recorded *I*–*t* transients nor examined, with the same experimental setup, the landing of NPs with diameters spanning the range claimed. Moreover, recent work shows that the electrochemical dissolution of single NPs is a somewhat more complex process.^26–30^ This makes a detailed analysis of NP oxidative impacts imperative, particularly as this methodology is now being extended to other systems, such as NP alloys^31^ and bioanalysis.^32^ It is further important to note that in many reports on impact coulometry, little information was given about signal amplification, sampling time and the data processing carried out to interpret NP impact current transients; these critical factors have only recently begun to be reported.^33,34^

Electrochemical oxidation of single Ag NPs of 10 nm in diameter has produced current magnitudes of 1 to 10 pA and anodic dissolution transient durations of 2–3 ms by using carbon fiber (CF)^20^ and Pt^34^ UMEs, but the use of a cut-off filter^20^ or signal averaging^34^ compromised the ability to probe short-time stripping events. In addition, the electrochemical oxidation of Ag NPs of 100 nm in diameter has led to peak currents spanning from 30–200 pA,^21^ on the one hand, to 0.5–10 nA,^18^ on the other, with an identical experimental configuration save for the use of different equipment to record the transients. This difference of almost 3 orders of magnitude in the peak currents for the anodic stripping of Ag NPs of the same size highlights the potential complexity of the stripping process, but raises significant questions on the effect of sampling rate and data filtering in the faithful acquisition of current transients. This important issue is only just beginning to be considered. For example, the effect on impact transients of the cut-off frequency of a Bessel filter has recently been reported.^35^ However, although the charge transferred in an impact process may be conserved irrespective of the bandwidth of the current follower,^33^ the features of the *I*–*t* transient are expected to be affected considerably by the filtering process.^36–38^ This is one of the major aspects of SNEI that we consider herein.

As an alternative to the use of UMEs, we have recently demonstrated that scanning electrochemical cell microscopy (SECCM) offers high sampling rates with low background current levels.^14^ This is due to the confinement of the electrochemical cell within a meniscus formed by a micropipette and the collector electrode. Hence, the SECCM platform has been used successfully for single-entity electrochemical measurements such as monitoring the electrodeposition of single NPs,^39,40^ electrochemical detection of single molecules^41^ and, most recently, the time-resolved detection of single NP electrocatalytic impacts,^14^ including surface oxide formation on noble material (Au) NPs.^42^ An advantage of the SECCM approach is that there is no need for an encapsulated collector electrode, so that a wide range of electrode materials can be used for this purpose, including materials with low noise characteristics.

In this work we use SECCM to study the electrochemical dissolution of single Ag NPs with diameters ranging from 10 to 100 nm upon impact on glassy carbon (GC) surfaces, as carbon collector electrode surfaces have been used most in previous studies (see ESI, Section S1†). For comparison, we have also studied 100 nm Ag NPs on an Au collector electrode. The SECCM setup allows us to obtain a peak-to-peak background noise of 4–5 pA with a current amplification time constant as low as 100 μs. Under these conditions, *I*–*t* transients are significantly different from, and more complex than, previous reports.^18,21^ We show that oxidative NP impacts are much more complex than considered hitherto. In particular, individual Ag NPs most typically dissolve through *multiple and repetitive impacts*, in which partial oxidation occurs, and for larger NPs overall *electrodissolution is often incomplete*. The resulting *I*–*t* traces can therefore not be used for NP sizing, as has been proposed. However, thorough examination of the recorded *I*–*t* curves provides important information on the near wall dynamics of NPs and their interaction with different surfaces during electrochemical dissolution processes, which is potentially highly valuable as an approach for understanding surface chemistry.

## 2 Experimental

### 2.1 Chemicals

NaNO_3_ (≥ 99.0%) and silver nanoparticles (Ag NPs) with five different sizes (diameter, *d* = 10, 20, 40, 60 and 100 nm) dispersed in sodium citrate aqueous solution were purchased from Sigma-Aldrich (St. Louis, MO). An aqueous supporting electrolyte of 50 mM NaNO_3_ was prepared with high purity water (Milli-Q, 18.2 MΩ cm resistivity at 25 °C). Citrate capped Ag NPs were chosen for this study, since they have been used in most previous single NP electrodissolution studies.^16–21,26,27,29,31^ Likewise, most previous studies have been carried out with citrate present in solutions (ESI, Section S1 and Table S1†).

### 2.2 Electrochemical dissolution of individual Ag NPs: experimental setup

Two different configurations (UME and SECCM) were tested in initial experiments to evaluate the most appropriate experimental setup and instrumentation for single NP impact studies. On the one hand, Au (25 μm diameter) and CF (7 μm diameter) disc UMEs were used in bulk solution to measure the background currents that arose from polarizing the working electrode at a potential (*E*) sufficiently positive to drive the oxidation of Ag NPs (*E* = 0.6 V *vs.* Ag/AgCl quasi-reference counter electrode, QRCE), in a 2-electrode arrangement. These measurements were performed in supporting electrolyte without Ag NPs. The QRCE has a potential of +188 mV *vs.* saturated calomel electrode (SCE) and is stable to within 3 mV over a 30 minute period (a larger period than the measurement time considered herein). The anodic dissolution process is thus driven strongly, which is the appropriate condition for sizing and analytical applications. The current amplification time constant of the electrometer was varied to study its effect on the measured background currents.

**Fig. 1:**
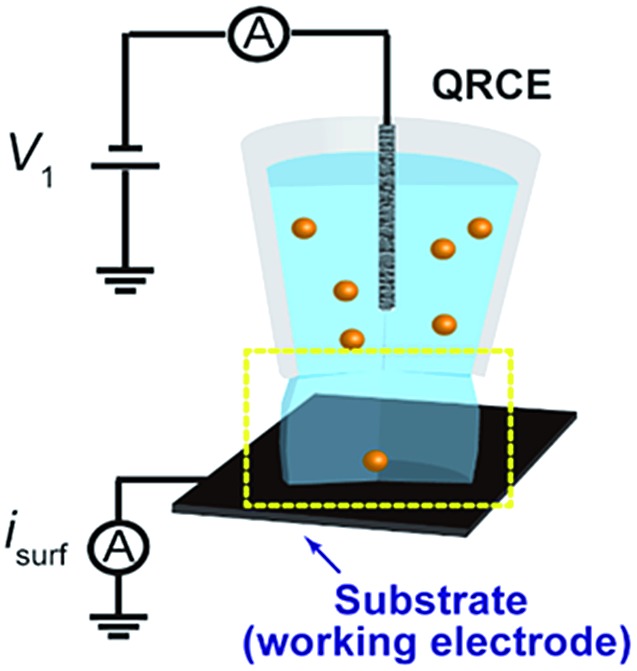
Schematic of the set up for SECCM NP impact experiments.

On the other hand, the SECCM setup (see Fig. 1)^43^ consisted of a single-barrelled pipette (aperture diameter of 5 μm) pulled from a borosilicate glass capillary utilizing a CO_2_-laser puller (P-2000, Sutter Instruments). The pipette was filled with a 1 : 1 solution of 50 mM NaNO_3_ and the as-purchased Ag NP solution. A AgCl-coated Ag wire was placed in the capillary and used as a QRCE. The pipette was mounted on a *z*-piezoelectric positioner (P-753.1CD LISA, PhysikInstrumente) and positioned on the surface of interest (meniscus-only contact) using an *xy*-piezoelectric positioner (P-622.2CD PIHera, PhysikInstrumente). The electrochemical cell and all positioners were placed in a Faraday cage with heat sinks and vacuum panels to minimize noise and thermal drift. Glassy carbon (GC) pieces (HTW-Germany) and Au UMEs (as for UME measurements) were used as the substrates to be comparable with the UME bulk measurements.

A potential of –0.1 V *vs.* Ag/AgCl QRCE was applied to the substrate as the pipette was approached towards it and the current from the substrate was recorded to detect the moment when the meniscus contacted the surface (current spike), but without contact from the pipette.^43^ This signal was used to stop the pipette movement. Once the meniscus was in contact with the substrate, the potential was switched to 0.6 V *vs.* Ag/AgCl QRCE, to observe the electrochemical dissolution of impacting Ag NPs.

A home-built potentiostat and electrometer were used throughout all measurements. Two home-built 8^th^ order brick-wall filter units were utilized to vary the time constant of the current amplifier (from 100 μs to 50 ms) and the data were recorded with an acquisition rate of 165 μs (sampling rate of 5 μs averaged 33 times). Data acquisition and fine control of all the instruments was achieved by using an FPGA card (PCIe-7852R) controlled by a LabVIEW 2013 interface. Data treatment was carried out with Igor Pro 6.37 (Wavemetrics).

The Ag NP size distributions were characterized by means of a Jeol 2100 Transmission Electron Microscope (TEM) operated at 200 kV, and by Dynamic Light Scattering (DLS) with a Malvern Zetasizer Nano ZS (Malvern Instruments Ltd., UK) (see ESI, Section S2†). Representative TEM images and estimations of NP concentrations and diffusion coefficients are also provided in ESI (Fig. S1, Tables S2 and S3,† respectively).

## 3 Results and discussion

### 3.1 Influence of the experimental setup, instrumentation and acquisition parameters on the background current (noise) level

In the absence of Ag NPs in solution, both GC and Au electrodes show little electroactivity with an applied potential of 0.6 V *vs.* Ag/AgCl. The current measured is due to background electrical and electrochemical noise inherent in the experimental configuration. These currents depend on the electrode surface area exposed to the electrolyte, cell design, the specifications of the current measuring instrumentation (amplification), the acquisition frequency (sampling rate or bandwidth) and the post-processing of the recorded data (filtering).^44^

As explained in the Introduction, it is mandatory to reach low background currents (≈pA) and high sampling rates (≈10 kHz), but these parameters are interrelated and there is a trade off.^36^ The time constant (*τ*_C_) of the setup is of high importance, particularly when the transient duration is of the same order of magnitude.^33,37,38^

**Fig. 2:**
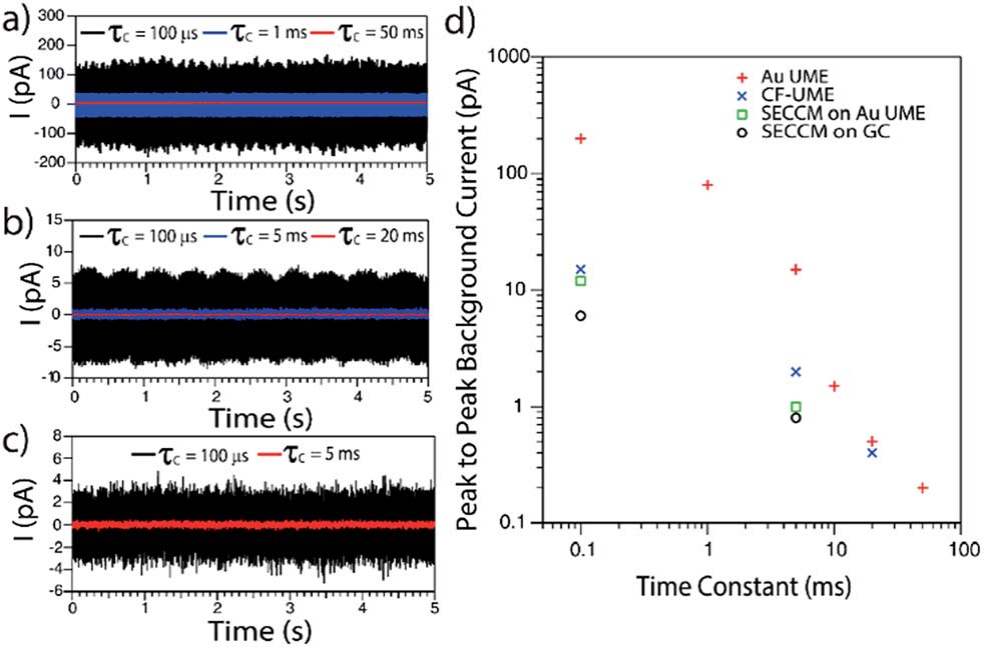
Representative *I*–*t* transients obtained during the polarization at *E* = +0.6 V *vs.* Ag/AgCl of a (a) Au UME, (b) CF-UME and (c) GC electrode in SECCM setup, with different amplification time constants. (d) Peak to peak background current *vs.* amplification time constant for different electrode materials and setups.

In order to evaluate the background currents for different time constant settings, electrode materials and electrochemical setups (UME and SECCM), *I*–*t* transients were recorded in solutions without Ag NPs during the application of *E* = 0.6 V *vs.* Ag/AgCl QRCE to the working electrode. Representative *I*–*t* transients are displayed in Fig. 2a–c. Peak to peak noise values have been measured and plotted against the amplifier *τ*_C_ in Fig. 2d.

As expected, decreasing *τ*_C_ results in an increase of the background current, irrespective of the electrochemical setup employed. Furthermore, and most importantly, it is confirmed that confining the electrochemical cell to a meniscus of several microns diameter (SECCM setup) leads to lower background currents than immersing an UME in a standard electrochemical cell.^45^ This is mainly due to the change in the collector electrode area (see ESI, Section S2 and Fig. S2†). It is also clear that for SECCM cells of similar area, GC gives lower background currents than Au as the collector electrode. In large part, this is due to less wetting (smaller contact area) of the GC substrate. We thus mainly focus on GC, although some comparative measurements on Au are also reported.

These data highlight some important considerations concerning the detection and analysis of single Ag NP stripping by impacts. If we consider the *I*–*t* response of a stripping event as a triangular spike, the charge consumed during such a transient could be approximated by *Q* = *I*_p_ *t*_e_/2, with *I*_p_ the maximum spike current and *t*_e_ the duration of the event. Then, for an Ag NP of *d* = 10 nm, the charge associated with its full electrochemical dissolution would be about 5 × 10^–15^ C. So, if a stripping event lasted for 10 ms, the peak current would only be 1 pA. Alternatively, peak currents of 10 pA would be expected if the events spanned just 1 ms. However, in order to accurately resolve such an *I*–*t* transient, the amplification time constant would need to be smaller than the event duration, otherwise the information of the *I*–*t* transient would be a convolution of the real process and the electronics of the instrument.^33,36–38^ The noise consequences of decreasing the time constant are evident in Fig. 2. It follows from this analysis that, to resolve events in the ms range, a *τ*_C_ of 100 μs would be essential. With these conditions, the SECCM configuration on a GC substrate is optimal.

### 3.2 Ag NP stripping using SECCM

Unless stated otherwise, the data presented in this section was obtained with a SECCM configuration with a current amplification time constant of 100 μs to allow an accurate resolution of ms to sub-ms stripping events. To examine the effect of the time constant on the analysis of single NP impacts, a comparison between *τ*_C_ = 100 μs and *τ*_C_ = 5 ms is made in Section 3.2.4.

#### 3.2.1 Qualitative description of *I*–*t* transients

**Fig. 3:**
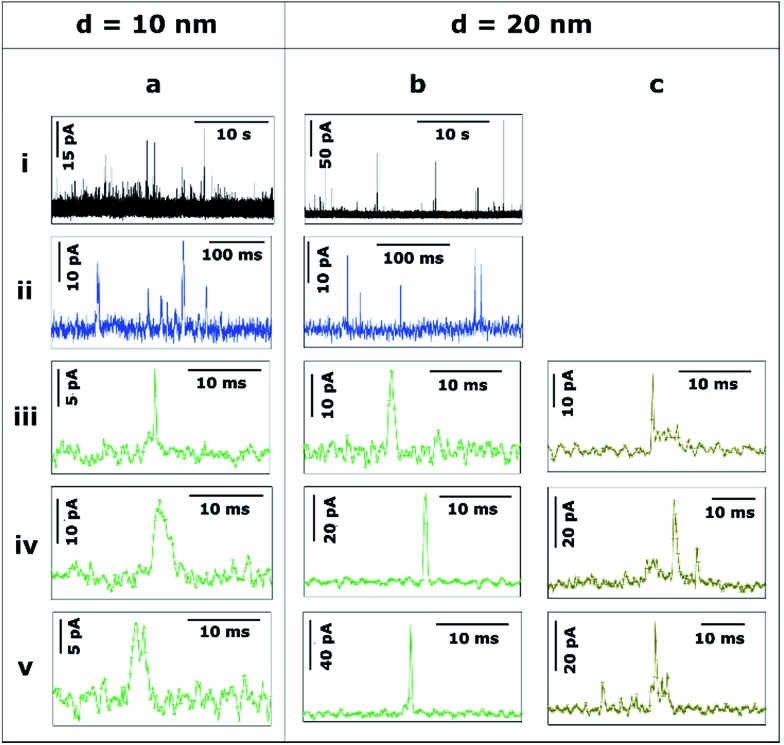
Representative current transients obtained by applying *E* = +0.6 V *vs.* Ag/AgCl to a GC electrode with a solution containing Ag NPs with nominal diameter of (a) 10 nm and (b and c) 20 nm. The numbers i–v refer to different cases discussed in the text.

**Fig. 4:**
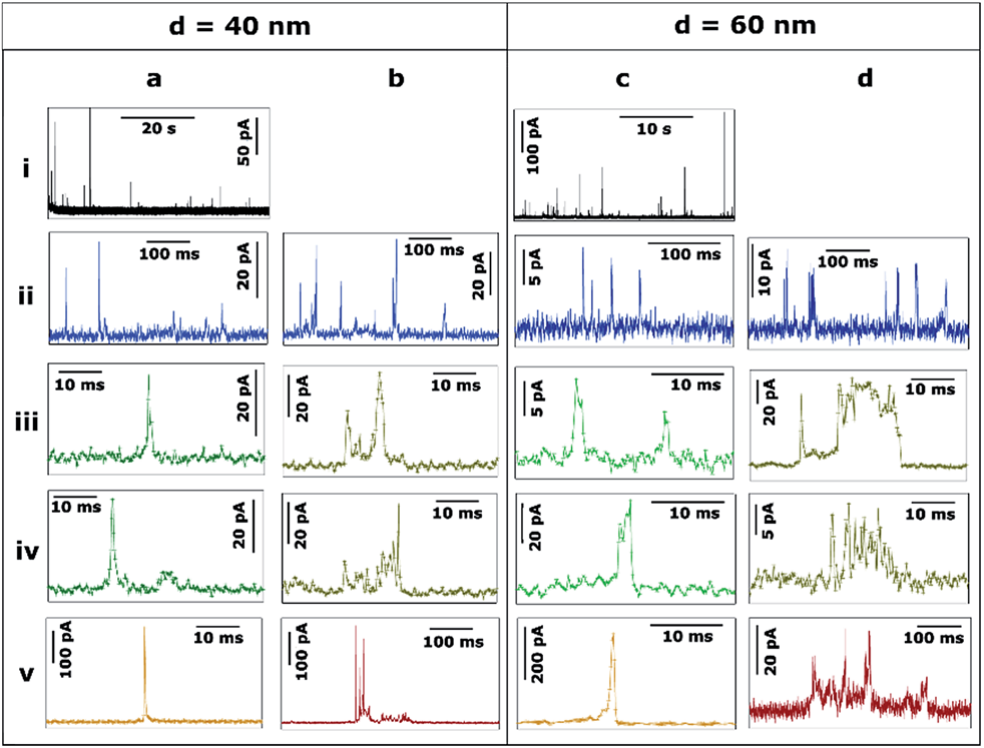
Representative current transients obtained by applying *E* = +0.6 V *vs.* Ag/AgCl to a GC electrode with a solution containing Ag NPs with nominal diameter of (a and b) 40 nm and (c and d) 60 nm. The numbers i–v refer to different cases discussed in the text.

**Fig. 5:**
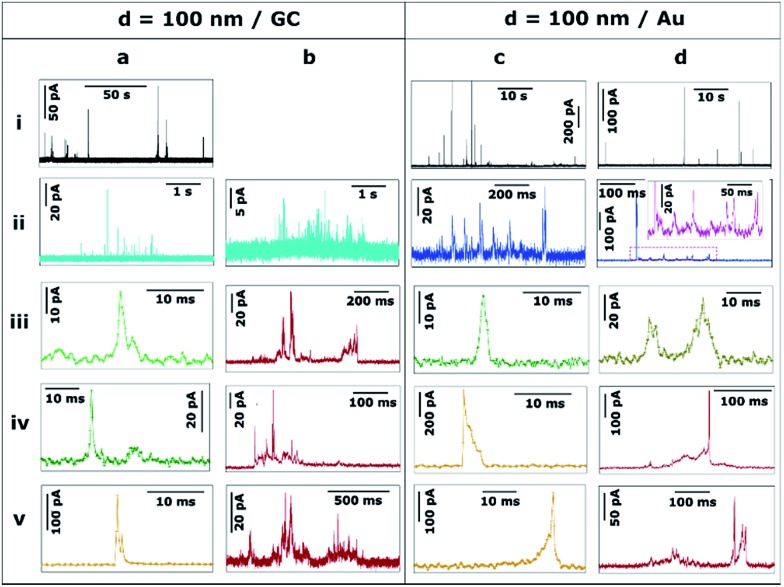
Representative current transients obtained by applying *E* = +0.6 V *vs.* Ag/AgCl to a (a and b) GC and a (c and d) Au electrode with a solution containing Ag NPs with nominal diameter of 100 nm. The numbers i–v refer to different cases discussed in the text.

Fig. 3–5 provide a summary of the main characteristic features of Ag NP stripping events based on the analysis of more than 2000 *I*–*t* impact transients.

Fig. 3 shows the transients during the electrodissolution of Ag NPs of *d* = 10 and 20 nm. The first column (Fig. 3a) shows characteristic events of the stripping of NPs of *d* = 10 nm. Although a few events with currents up to 40–80 pA can be observed (3a.i; black), the most representative population of events comprises peak heights less than 15 pA (3a.ii; blue) and sharp durations of 0.3 to 5 ms (3a.iii, 3a.iv, 3a.v; green).

The second and third columns (Fig. 3b and c) show the characteristic features upon stripping of NPs of *d* = 20 nm. The characteristics are similar to *d* = 10 nm: fast and sharp events of duration shorter than 5 ms (3b.iii, 3b.iv, 3b.v), but with slightly higher maximum currents (10–20 pA). However, there are a few longer events (*t* = 5–20 ms) with an irregular, saw-tooth profile (3c.iii, 3c.iv, 3c.v; brown). Further interpretation of these features is given in Section 3.2.2. Evidently, the electrodissolution processes on green and brown *I*–*t* transients must be different from each other.

Fig. 4 shows the transients during the electrodissolution of Ag NPs of *d* = 40 and 60 nm. In both cases, the appearance of isolated events of much larger maximum current, compared to Fig. 3, is evident (4a.i, 4c.i). This response is shown in detail (4a.v and 4c.v; orange) and evidences that the duration of such orange events (*t* = 1–3 ms) is similar to that of green events (4a.iii, 4a.iv, 4c.iii, 4c.iv) but with much higher current (*I* > 100 pA). The charge consumed in these events approaches, but is not, full stripping, corresponding to Ag NPs of *d* = 30 nm (4a.v) and *d* = 47 nm (4c.v). More interestingly, green (*t* < 5 ms; *I* = 5–30 pA) low current fast events and brown (*t* = 5–10 ms; *I* = 5–40 pA) low current, saw-tooth, longer events are still prevalent. These events are sometimes isolated (4a.ii, 4a.iii, 4c.iv) and sometimes grouped in bundles (4a.iv, 4c.ii, 4c.iii). Most importantly, in none of these cases does the charge consumed during single events account for the stripping of entire NPs and hence represents only a partial stripping process.^27–29^ It should be noted that when the NP diameter increases from 20 to 60 nm, the occurrence of such bundles of low current (green and brown) events is more frequent (28% and 58% of the total charge for *d* = 20 nm and *d* = 60 nm, respectively). Furthermore, contrary to the cases of *d* = 10 nm and *d* = 20 nm, for *d* = 40 and *d* = 60 nm, very long saw-tooth events are also present (Fig. 4b.v and 4d.v; red) with durations that span to 100–500 ms and peak currents that span from a few tens to hundreds of pA. In these specific cases, the total charge associated with these events may indicate total stripping of large particles as the inferred diameters are of *d* = 64 nm (4b.v) and *d* = 70 nm (4d.v), respectively. Further quantitative analysis is reported in Section 3.2.2.

Ag NPs of *d* = 100 nm were landed on both GC (Fig. 5a and b) and Au (Fig. 5c and d) collector electrodes. With GC as the collector electrode, Fig. 5a.i shows 140 seconds of the current response. Peak currents range from 10 to 100 pA. Note that the event frequency is low because the total concentration of Ag was maintained for the different sized particles in these studies (see ESI, Section S2†). This was advantageous because, as we show in this section, the stripping process became increasingly complex – and could be of much longer duration – as the NP size increased. We thus achieved conditions where the mean primary first pass diffusion frequency, *f*_NP_, was small (average time between first impact of NPs of 40 s in the case of *d* = 100 nm NPs) so that a sequence of complex events on (much) shorter time scales could reasonably be assigned to a single NP (ESI, Table S3†).

A closer examination of the current transients reveals 3 distinct types of events, which suggest 3 underlying stripping processes. On the one hand, bundles of fast events with low currents (≤ 20 pA) are shown in Fig. 5a.ii and 5b.ii (light blue). These bundles span for a few seconds in total but are constituted by individual green events (details in 5a.iii and 5a.iv) that last only for a few ms each. The charge consumed in such individual events is of the order of 10^–15^ to 10^–13^ C that would correspond to NPs of *d* = 10–20 nm. Such small particles are absent in these measurements as confirmed by TEM and DLS (see ESI, Section S2†). Thus, each of these events represents the partial stripping of a small fraction (0.1–2%) of the same particle of *d* = 100 nm. We consider it most likely that this is due to the temporary detachment of a NP from the surface after partial stripping, followed by re-engagement and a subsequent partial stripping event. A similar interpretation could be drawn for the experiments carried out with NPs of *d* = 40 and *d* = 60 nm (Fig. 4b.ii, c.ii and d.ii). The repetitive engagement and detachment of NPs with a collector electrode has been previously demonstrated for ruthenium oxide NPs impacting on HOPG during catalytic amplification of hydrogen peroxide oxidation, and can be revealed provided that the time constant is sufficiently short.^14^ Additionally, the ready detachment of Ag NPs during electrodeposition has also been shown.^40^

Furthermore, short, sharp, high-current orange (5a.v) and long low-current, saw-tooth red (5b.iii, 5b.iv, 5b.v) events spanning hundreds of milliseconds are found, with a higher occurrence than for smaller NPs. Although large amounts of charge are consumed in these cases, the whole NP is not necessarily oxidized to Ag^+^ cations, since the charge consumed on events 5b.iii, 5b.v, 5b.iv and 5a.v would correspond to *d* = 90 nm, *d* = 98 nm, *d* = 80 nm, and *d* = 30 nm, respectively. Whereas full particle stripping probably occurs in the two first cases, only 50% and 3% of a NP of *d* = 100 nm are dissolved in the latter two examples.

Fig. 5c and d show characteristic transients on a Au electrode. At first sight, the features of these transients are similar to *d* = 100 nm Ag NPs landed on GC (Fig. 5a and b), but with some differences discussed here and in Section 3.3. On the one hand, the bundles of small charge events are constituted by individual (green and brown) events, whose duration is slightly longer on Au than on GC. Such individual events span from less than 5 ms (5c.iii) up to 30 ms (5d.iii). On the other hand, a typical bundle is a few hundred ms long, which is less than for GC (few seconds).

Similar to GC, the most prevalent types of stripping events on the Au collector electrode are short, sharp, high-current orange (5c.iv, 5c.v) and long low-current, saw-tooth red (5d.iv, 5d.v) events. However, there are some subtle differences. Using a Au electrode, sharp orange events span for 5–15 ms, approximately twice the duration as on GC. These sharp events are asymmetric having either a very sharp increase in current followed by a longer decay (5c.iv) or *vice versa* (5c.v). The first case represents a NP engaging quickly at the collector electrode and being held back as it attempts to disengage.^14^ The second case embodies a NP gradually engaging with the electrode and sticking, and then quickly detaching after partial stripping. It should be noted that the charge consumed in both cases would represent the partial stripping of fractions of 18% and 22% of a particle of *d* = 100 nm. This asymmetry is not only noticeable on Au, but can also be seen in GC where sharper current increase (5a.v) or decrease (4c.v) can also be detected, although these events occur more frequently on Au (35%) than on GC (8%).

Saw-tooth red (5d.iv, 5d.v) events spanning hundreds of ms are also found on Au. They represent only 25% of the total charge (54% for GC). Such events attain currents up to hundreds of pA. Again, although large amounts of charge are also consumed in these cases, total stripping cannot always be guaranteed, as while the charge consumed on event 5d.iv corresponds to *d* = 100 nm, that on 5d.v represents *d* = 80 nm (or 50% of a Ag NP of *d* = 100 nm).

#### 3.2.2 Quantitative analysis of *I*–*t* transients

It is worth reemphasizing that the measurement of the charge associated with the electrochemical dissolution of single NPs upon impact onto a collector electrode has been claimed to allow the sizing (including determining the size distribution) of NPs with diameters from 6 to 100 nm with a resolution comparable to TEM.^20,21^ The qualitative analysis of the features of the *I*–*t* transients reported in Section 3.2.1 suggests otherwise. In this section, a quantitative analysis of the main types of current transients is presented, which confirms the deductions made from our qualitative survey of the wide range of *I*–*t* morphologies that occur in NP impacts.

**Fig. 6:**
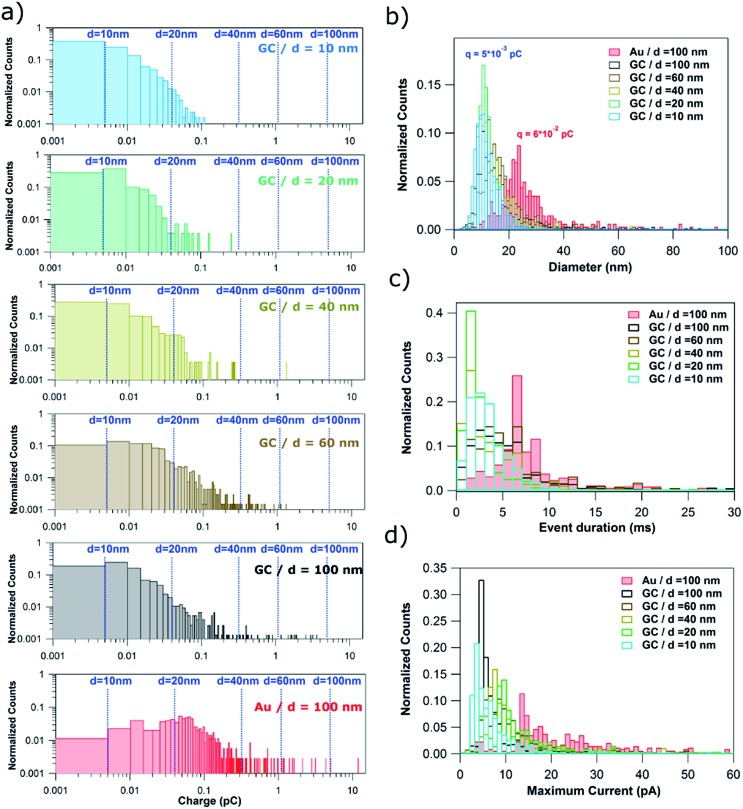
(a) log–log plot of the charge histograms for single events recorded during the stripping of Ag NPs with nominal diameter of 10, 20, 40, 60 and 100 nm on GC and 100 nm Ag NPs on Au electrodes. Histograms of the (b) equivalent NP diameter (assuming dissolution of whole NP), (c) event duration and (d) maximum current.

Fig. 6a shows normalized histograms of the charge consumed by each single event (current spike) for the stripping of NPs of *d* = 10, 20, 40, 60 and 100 nm on GC and *d* = 100 nm on Au. The charge data span 5 orders of magnitude and so histograms are displayed as a log–log plot in order to better visualize all the data. The dashed blue lines correspond to the charge associated to the electrochemical oxidation of Ag NPs of ideal spherical shape with diameters of 10, 20, 40, 60 and 100 nm.

When using GC as the collector electrode, the great majority of single events consume charges ranging from only 1 × 10^–15^ to 5 × 10^–14^ C, independent of NP size. Such charges would be equivalent to NPs of *d* = 10–20 nm. Consequently, such events could represent (close to) full NP stripping when *d* ≤ 20 nm. However, the upper limit, a charge of 5 × 10^–14^ C, represents only 15%, 5% and 1% of NPs with *d* of 40, 60 and 100 nm, respectively. This confirms that for particles of this size, most of the *I*–*t* transients correspond to partial stripping of a small fraction of an impacting NP, followed by the release of the remaining NP. Events associated with the full stripping of large NPs are extremely rare. For the Au collector electrode, the charge histogram peaks around 6 × 10^–14^ C, higher than for GC (5 × 10^–15^ C). Thus, the charge consumed in single events is more dependent on the collector electrode material than on the NP size. This difference can be explained by the higher affinity of Ag towards Au^46^ than towards GC.^47^

The charge consumed can be converted to an equivalent diameter using Faraday's law. Fig. 6b shows the histograms of equivalent NP diameters inferred from the charge histograms in Fig. 6a. The same conclusion can be drawn: complete particle stripping on GC occurs only for particles with *d* = 10 nm. The histograms for NPs of larger diameter give an incorrect NP size distribution. Again, the influence of the collector electrode material can be clearly seen as the equivalent diameter histogram peaks at 25 nm for Au, most likely due to the stronger interaction and longer duration that Ag NPs spend on the Au surface (Fig. 5 and associated discussion above).

In Fig. 3–5, it was shown that many events span over hundreds and thousands of milliseconds (brown and red events). Such events are consistently longer and more common for larger NP size (from 10% of all events for *d* = 20 nm to 55% when *d* = 100 nm). This can be inferred from the complete event duration histograms (Fig. 6c, with data also shown with a log–log scale in Section S3, Fig. S3, of the ESI†). In Fig. 6c, the *x*-axis stops at 30 ms to better visualize the event duration distributions for the shorter times, as these are the most prevalent (green and orange events in Fig. 3–5). Fig. 6c shows a slight increase with NP size in the mode of the histograms for GC, being 1–2 ms for *d* = 10, 20 and 40 nm, and 3–4 ms for *d* = 60 and 100 nm. The distinct difference between Au and GC collector electrodes for NPs of *d* = 100 nm is very clear, with the histogram for Au peaking at around 7–9 ms. It is worth reemphasizing that such subtle differences can only be attained using sub-ms amplification time constants, as in this study.

In Fig. 3–5, it was evident that some events reached hundreds of pA (orange and some red events), particularly for larger NP sizes, although such events are rather rare. This can be inferred from the complete maximum current histograms, shown with a log–log scale in Fig. S4 of Section S4 of the ESI.† A histogram of the maximum currents recorded for single events is also displayed in Fig. 6d. The *x*-axis is cut off at 60 pA, as currents up to this value represent the majority of events (green, brown and some red events in Fig. 3–5). Fig. 6d highlights that for GC most of the events are between 4 and 10 pA, whereas for Au as the collector electrode, most of the events attain currents between 14 and 30 pA.

Given that the consumed charge (Fig. 6a), the event duration (Fig. 6c) and the maximum currents (Fig. 6d) are all dependent on the collector electrode, electrochemical dissolution of single NPs upon impact can be used to highlight differences in the physicochemical interactions and electron transfer reactions between colloidal NPs and different surfaces. That the electro-oxidation, as manifested in the *I*–*t* characteristics, is very different on these two electrodes, with further elaboration below, is also very good evidence for the proposed mechanism, involving transient interactions and partial electro-oxidation of Ag NPs at the collector electrode surface. Were the behaviour due to other processes, such as the possible formation of Ag_3_Cit (for example), one would expect the same behaviour for the same sized NPs on different collector electrodes, and this is not the case. As shown in the voltammetric data in the ESI (Fig. S5†), citrate inhibits the electrodissolution of Ag (most likely by adsorption), but there are no discrete passivation events, even in constant potential *I*–*t* measurements, with high time resolution and under conditions where the near-electrode concentrations of Ag^+^ are comparable to, and in excess of, those pertaining to the studies herein and over time scales that are orders of magnitude longer.

#### 3.2.3 Quantitative analysis of the time elapsed between consecutive events

Another parameter that can be deduced from a quantitative analysis of the *I*–*t* transients is the event frequency or its inverse – the time between two consecutive events. In previous NP impact studies, the event frequency has been found to be of the order predicted assuming a single pass diffusive flux of NPs^11,16,48^ or slightly lower.^4,12,49^ However, we recently showed that the measured frequency can actually be several orders of magnitude larger than expected based on simple diffusion, due to the rapid and repetitive trapping and release of a single NP upon impact.^14^

**Fig. 7:**
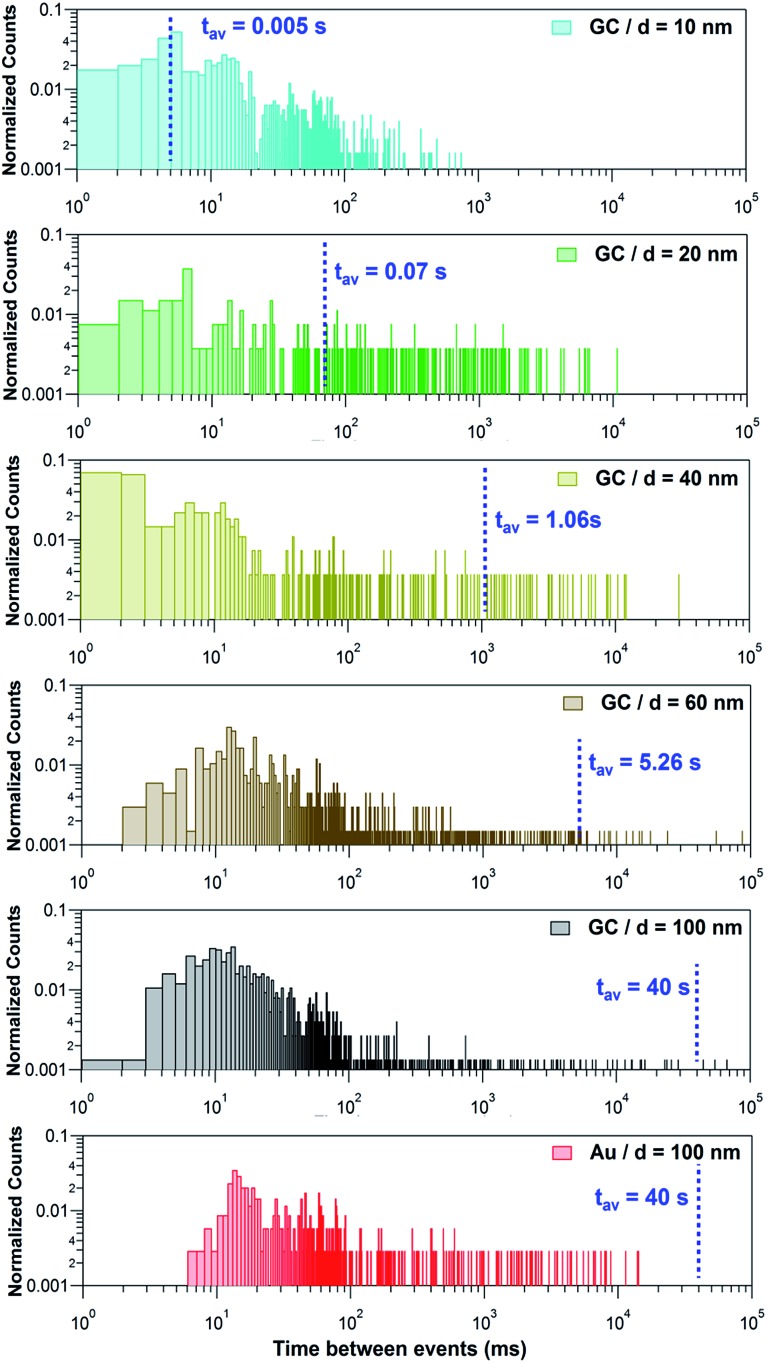
log–log plot of the time elapsed between consecutive events recorded during the stripping of Ag NPs with nominal diameter of 10, 20, 40, 60 and 100 nm on GC and Au electrodes. The blue dashed line represents the estimated average time between two events if each NP was fully stripped in one single event.

Fig. 7 shows histograms of the peak to peak time elapsed between two consecutive events for the Ag NPs studied on the 2 different collector electrodes. This magnitude spans over several orders of magnitude, from milliseconds to several seconds and so histograms are shown with a log–log scale. The blue dashed lines correspond to the estimated average time that would be elapsed between two consecutive events, based on the diffusive flux (see ESI, Section S2†) if each NP was fully dissolved in one single impact. Such average time is 1/*f*_NP_, with *f*_NP_ the estimated impact frequency assuming single pass NP diffusion. The measured concentrations and estimated diffusion impact frequencies are given in Section S2 and Table S3 of the ESI.†

If NPs were fully stripped in a single event, these histograms would show a normal distribution centred at the estimated average time between events. However, this is only the case for *d* = 10 nm. Instead, it can be seen that, for *d* ≥ 20 nm there is a much higher proportion of events that occur with a frequency (shorter time between events) that is higher than expected for single pass diffusion and that the higher frequency of impacts becomes more significant as the NP size increases. This is a confirmation that, except for NPs of *d* = 10 nm, larger NPs undergo multiple partial stripping impacts with the collector electrode. This is in line with the equivalent diameter histograms shown in Fig. 6b and with the fact that only for *d* = 10 nm, is there a single type of *I*–*t* response (Fig. 3a, only green events).

For *d* ≥ 20 nm, it is evident that, on the one hand, most of the events are separated in time by less than 50 ms. On the other hand, the separation between some events may span up to 100 s. It is reasonable to propose that the first distribution corresponds to repetitive partial stripping events of the same NP, in which the NP size is reduced in a series of ‘bites’ which may, or may not, ultimately lead to complete dissolution (see Section 3.2.5, below).^14,50^ This mechanism corresponds to the event bundles depicted in Fig. 4b.iv, d.ii, d.iii and 5a.ii, b.ii, c.ii, d.ii and d.iii. The longer timescale events relate to the time that elapses between two separate NPs diffusing from the bulk solution towards the electrode.

For *d* = 20 and *d* = 40 nm, the expected time between two NPs impinging on the electrode falls reasonably in the centre of the second distribution, corresponding to the first impact that a NP makes with the collector electrode. Hence, this would also mean that, although undergoing several partial events, NPs of this size could eventually be fully stripped, in several discrete stripping events. However, for NPs of *d* = 60 and *d* = 100 nm, because the overwhelming majority of the inter-event times are much shorter than the diffusion flux time, a NP moves back and forth many times from the solution to the near-wall region, and undergoes a series of partial stripping events on each arrival.

A further striking feature is the difference in impact times between the two collector electrodes for 100 nm Ag NPs. When Au is the collector electrode, the time between events is > 7 ms, but on the GC collector electrode, many events have a much shorter timescale. This is because Ag NPs make much weaker (shorter time) contact with GC,^40,51,52^ leading to a higher frequency of attachment (impact)-detachment events compared with Au as the collector electrode.

#### 3.2.4 Effect of the amplification time constant in the analysis of stripping events

**Fig. 8:**
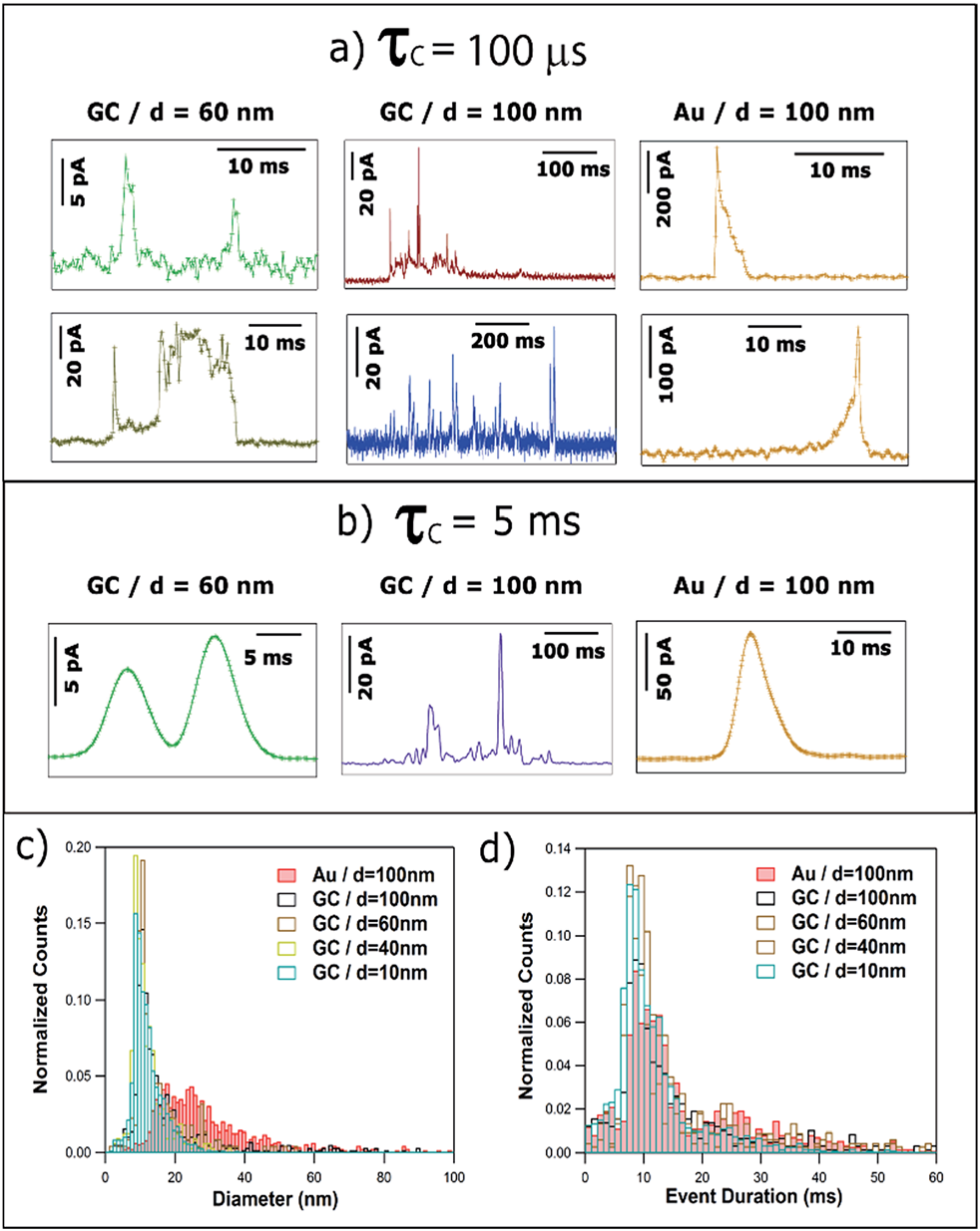
Representative current transients obtained with (a) *τ*_C_ = 100 μs and (b) *τ*_C_ = 5 ms, by applying *E* = +0.6 V *vs.* Ag/AgCl to a GC electrode with a solution containing Ag NPs with nominal diameter of (left) 40 nm and (middle) 100 nm, and to a Au electrode with a solution containing Ag NPs with nominal diameter of 100 nm (right). Histograms of (c) equivalent NP diameter (assuming complete dissolution of NPs) and (d) event duration, both for *τ*_C_ = 5 ms.

We briefly examined the effect of the time constant on the current response for two reasons: (i) a number of studies in the literature, carried out with commercial potentiostats, appear to have employed a much longer time constant than considered for our studies;^17–19,24,53^ and (ii) a long time constant will lead to a merging (summing) of discrete events, and it was worth testing if this allows the NP size to be recovered. Fig. 8a shows selected *I*–*t* transients, recorded with *τ*_C_ = 100 μs, that display distinctive features (taken from Fig. 3–5 and discussed in Section 3.2.1). For comparison, Fig. 8b shows representative *I*–*t* signals taken under the same conditions, but with *τ*_C_ = 5 ms. The events correspond to 60 nm Ag NPs (left hand side) and 100 nm Ag NPs (centre) on GC, and to 100 nm Ag NPs on Au (right hand side). Increasing the time constant results in the signal being smoothed and is detrimental to the correct interpretation of NP landing events, which we have discussed in detail above. On the left hand side, it can be seen that the bundles of green events and saw-tooth brown transients become indistinguishable with *τ*_C_ = 5 ms. When the size of the impacting NP is larger, longer red saw-tooth events and blue event bundles are clearly distinguished with *τ*_C_ = 100 μs, whereas it is not possible to distinguish them from each other when *τ*_C_ = 5 ms, as displayed in the centre of Fig. 8b. Furthermore, on the right hand side, it is seen that when orange sharp events are detected with *τ*_C_ = 100 μs, an asymmetry is evident, allowing us to differentiate between fast current increase – gradual current decrease and *vice versa*, discussed above. A higher *τ*_C_ results in an altered measured transient^37,38^ that does not allow this discrimination.

The quantitative analysis of more than 4000 events recorded with *τ*_C_ = 5 ms is summarized in Fig. 8c and d. On the one hand, the equivalent particle size distribution, assuming that NPs are anodically dissolved in one impact, is shown in Fig. 8c and is very similar to that for *τ*_C_ = 100 μs (see Fig. 6b). This similarity is due to the fact that increasing the amplification time constant results in an altered *I*–*t* profile (much smaller current and longer duration) but the charge transferred is well conserved.^33^ The computed average diameters increase slightly for *τ*_C_ = 5 ms because events that are separated by very short times (*t*_sep_ < *τ*_C_) would be identified as one single event of larger charge. Fig. 8d shows the histogram of the duration of single events. The mode of the histograms is between 9 and 12 ms, and is independent of NP size and collector electrode material. This is in contrast with data reported for *τ*_C_ = 100 μs (see Fig. 6c) that showed a clear difference between the average event duration for GC (1–4 ms) and Au (7–9 ms). It is worth reemphasizing that these apparently subtle differences are of great importance in understanding the dynamics of NPs in the vicinity of polarized surfaces. Studies with the 5 ms time constant would conclude that the collector electrode surface chemistry was not important, whereas the shorter time constant reveals the significance of the surface chemistry in determining the nature of the impact event. It is further important to note that, even using slow time constants, similar to the ones in previous reports,^20,21^ NPs of *d* ≥ 20 nm do not undergo complete dissolution upon impact,^27–29^ and such data cannot be used reliably for NP sizing.

### 3.3 Electrochemical dissolution mechanisms of individual NPs

**Fig. 9:**
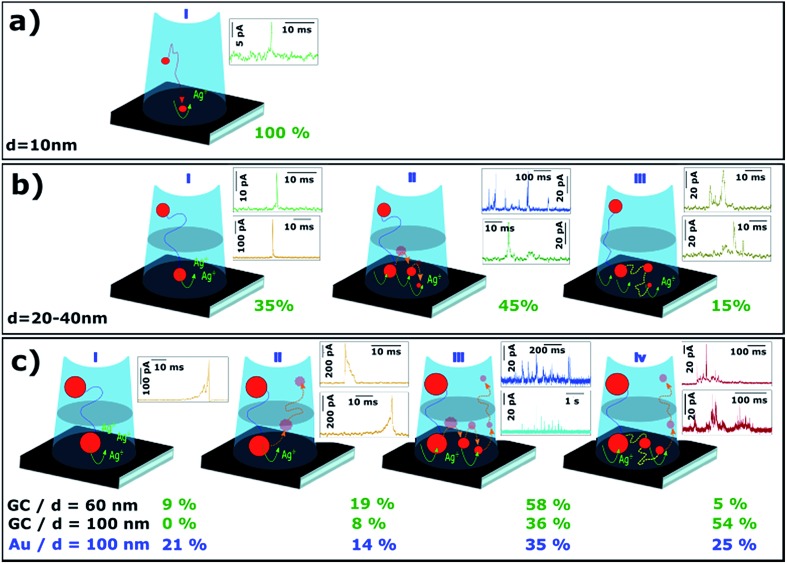
Schematic of different processes of electrochemical dissolution of Ag NPs with nominal diameters of (a) 10, (b) 20, 40 and (c) 60, 100 nm.

Since both Au and GC surfaces are heterogeneous and there is a certain dispersion in size and shape of NPs of a nominal size, variability in the *I*–*t* responses within a particular experiment is expected, as each event probes the interaction of a single NP with the region(s) of the collector electrode where it lands. Surface chemistry has been shown to have a significant effect on the residence time (and hence interaction) of NPs with collector electrode surfaces (overall comparison of the response on GC and Au collector electrodes), although it should be noted that electron tunnelling between an electrode and an adsorbed NP is relatively immune to passive layers (*e.g.* adsorbed impurities), unless the layer becomes too thick, with a dependence on the NP size.^54^ Fig. 9 shows the most recurrent morphologies for *I*–*t* transients, from which aspects of the dissolution mechanisms can be inferred (as a function of NP size). The percentage next to each scheme marks the proportion of that type of event, calculated as the ratio of the charge passed for events with that particular characteristic to the total charge consumed by events of all types.

For particles of *d* = 10 nm, virtually 100% of the recorded events have maximum currents between 4 and 40 pA, and durations between 1 and 10 ms (80% have durations between 2 and 4 ms). This type of event has been coded as green event in earlier sections of the article. The charge consumed in these events is in very good agreement with that required for the electrodissolution of a NP of *d* = 10 nm (see Fig. 6a and b). Hence, on the timescale accessible for these measurements, the electro-oxidation of small NPs of *d* = 10 nm, upon impact on GC, essentially occurs in a single event (Fig. 9a).

For particles of *d* = 20 and 40 nm, there are at least three scenarios. The charge passed in some green and orange events is consistent with the stripping of an entire NP, but occurs in only 35% of the cases (Fig. 9b.i). The most common scenario for green events (Fig. 9b.ii) is that the charge accounts for just a fraction of that needed to fully dissolve the impacting NP. This is also evident when one considers the time elapsed between consecutive events, which is much shorter than expected if each NP were to impact only once by diffusion (see Section 3.2.3). In the main, NPs of *d* ≥ 20 nm require several impacts to be dissolved completely. A possible scenario is that, after a first partial stripping event, the NP leaves the tunnelling (charge transfer) region, but remains in the vicinity of the surface due to near-wall hindered diffusion.^14,50^ There is then a high chance for such a partially dissolved NP to impact on the collector surface again and undergo another (partial) stripping event. The analysis of the time between consecutive events (see Section 3.2.3, Fig. 7) points towards NPs in this size range being eventually stripped in multiple events before leaving the near-wall region.

A third scenario is displayed in Fig. 9b.iii. Although less frequent, 15% of the charge consumed during the stripping of NPs of *d* = 20 or 40 nm occurs in longer events that display a saw-tooth shape, *i.e.*, several current maxima and minima instead of a single current peak. These events have been coded brown and have durations of tens of ms, with the current never dropping to zero. However, it must be noted that each data point represents the average current transferred during 165 μs, and the NP could move away from the electrode and back again in this time period. In essence, a saw-tooth *I*–*t* profile indicates that the NP is not firmly attached to the substrate during the stripping process. For this scenario, and for NPs of this size range, the charge consumed during these long brown events appears to be consistent with the electrochemical dissolution of entire NPs.

For larger diameters (*d* = 60 and 100 nm), the *I*–*t* transients also show several different scenarios. On the one hand, there are short and isolated current transients that reach several hundreds of pA and last less than 30 ms (orange events), but these events only account for 28% and 8%, respectively, of the total charge for 60 nm and 100 nm Ag NPs on GC. This scenario is displayed in Fig. 9c.i and 9c.ii. In relatively few cases (9% for *d* = 60 nm) is the amount of charge consumed during these events close to that required for entire NP electrodissolution (9c.i). In most cases, the charge passed corresponds to only a fraction of the landing NP, with no pre- or after transient signal. This means that after partial stripping, the NP drifts away into the bulk solution (Fig. 9c.ii). Such a NP could impact with the electrode again after a longer time has passed, generating another *I*–*t* transient. This scenario is in agreement with the conclusions from the analysis of the time between consecutive events (Fig. 7 and discussion above). Still, if only these events (*I*_Peak_ > 100 pA) were taken into account, the calculated average NP diameter would be 39 nm and 47 nm for Ag NPs of nominal diameters of 60 nm and 100 nm, respectively, landing on GC. Alternatively, on Au, an estimated diameter of 60 nm would be obtained for Ag NPs of 100 nm nominal diameter. This reemphasizes that impact coulometry cannot provide an accurate measurement of the size of NPs in solution.

Inspection of the orange transients leads to further insights into the stripping processes. Independent of NP size, and of whether partial or full stripping takes place, such sharp events are asymmetric and can be classified in two sub-types. In some cases, a very sharp increase in current is followed by a longer decay (Fig. 5a.v and c.iv) and *vice versa* (4c.v, 5c.v), as we discussed above (see Section 3.2.1). These events last approximately twice as long on Au as on the GC collector, indicating that Ag NPs tend to stay for longer time in the tunnelling region close to Au than to a GC electrode due to a stronger interaction between Ag and Au.^46^

The majority of *I*–*t* traces for 60 and 100 nm diameter NPs are comprised of short, low-current events (9c.iii) or long, irregular and saw tooth events (9c.iv). These cases are analogous to those shown in Fig. 9b.ii and 9b.iii respectively. The bundles of short events span up to few seconds whereas saw-tooth events can last up to hundreds of ms. Larger NPs with smaller diffusion coefficients are less likely to leave the near-wall region after partial stripping (9c.iii) and tend to stay within the tunnelling region (9c.iv) for longer times.

To reiterate, the relative occurrence of the different scenarios (Fig. 9c) depends on the collector electrode material. For *d* = 100 nm, the proportion of 9c.i and 9c.ii scenarios is higher for Au (35%) than for GC (8%). On the other hand, longer saw-tooth events (scenario 9c.iv), which reflect weak interaction between the NP and collector electrode, are more frequent on GC (54%) than on Au (25%).

### 3.4 Special case: periodic current transients

**Fig. 10:**
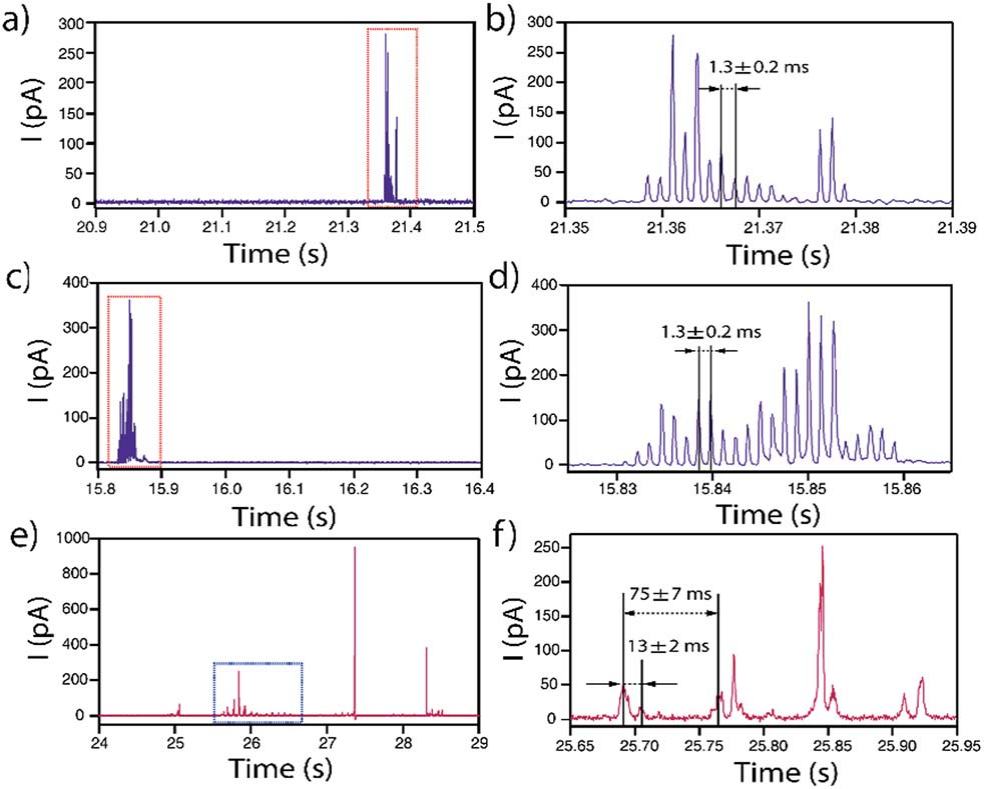
Representative periodic *I*–*t* transients obtained by applying *E* = +0.6 V *vs.* Ag/AgCl to a GC with a solution containing Ag NPs with nominal diameter of (a and b) 40 nm and (c and d) 60 nm, and to Au with a solution containing Ag NPs with nominal diameter of (e and f) 100 nm.

In some occasions, isolated current profiles displaying a periodic pattern were found. Fig. 10 shows three examples obtained for NPs of *d* = 40 nm (a and b), *d* = 60 nm (c and d) on GC and *d* = 100 nm on Au (e and f).

In the case of GC, for both *d* = 40 and *d* = 60 nm, the periodicity of the current profile is so ideal (within the resolution of the measurement) that the separation between current peaks is 1.3 ± 0.2 ms (8 data points). The possibility that this could be an electronic artefact can be discarded as such events are recorded during long *I*–*t* acquisitions that mainly display events as shown in Fig. 3–5. It must be noted that the charge consumed during the whole duration of these periodic events is equivalent to NPs of *d* = 44 nm and *d* = 60 nm, respectively, which indicates that the NPs are more or less completely consumed in a series of ‘bites’.

When the substrate is Au instead of GC, the repeated feature for *d* = 100 nm is not a single peak but a doublet, which repeats every 75 ± 7 ms. The two peaks that constitute the doublet are separated by 13 ± 2 ms. Interestingly, the charge consumed during the whole duration of this periodic event is equivalent to a NP of *d* = 94 nm, indicating that close to full NP stripping also occurs.

**Fig. 11:**
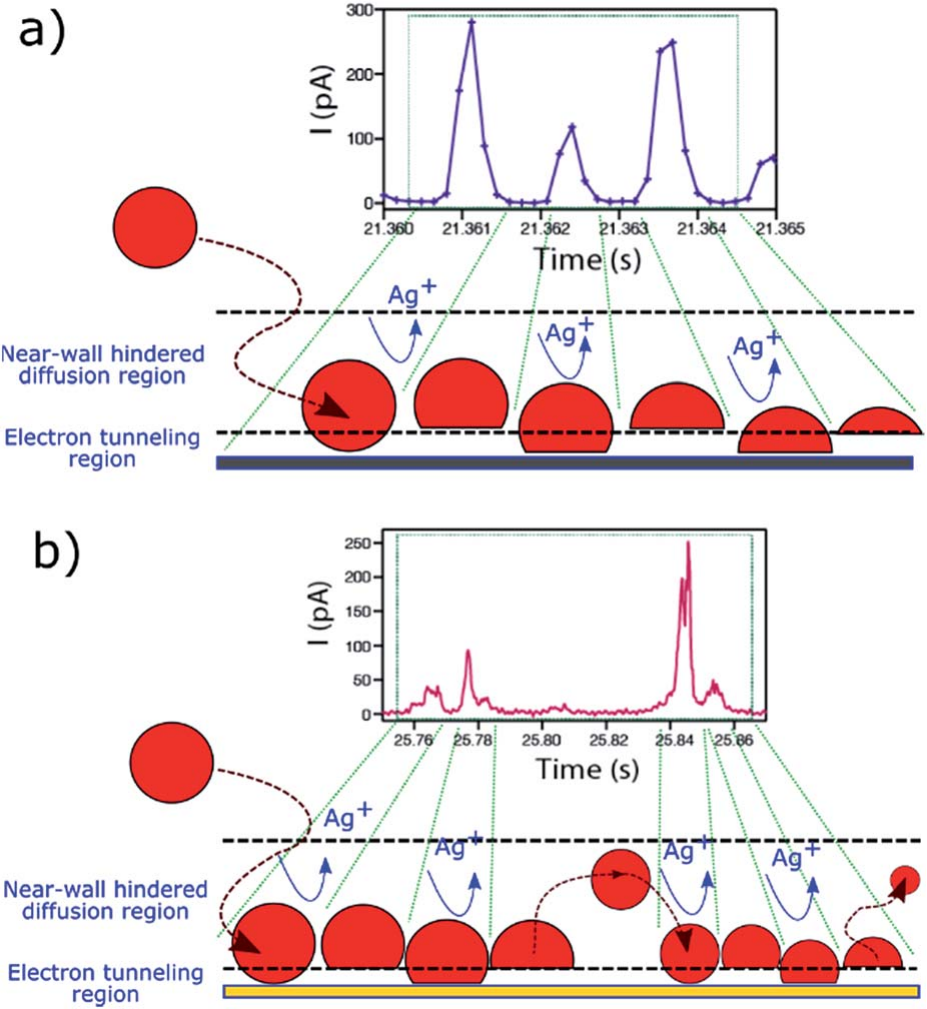
Periodic *I*–*t* patterns (from Fig. 10) and schematic representation of the electrochemical dissolution mechanism of Ag NPs on a (a) GC and on (b) Au substrate.

The fact that these oscillatory phenomena are very infrequent (<1%), implies that rather special conditions need to be fulfilled to give rise to this *I*–*t* behaviour. The NP is cycled to and from the collector with a close to constant periodicity, which is different on Au and GC substrates. These specific NP dynamics near the electrode cannot be caused by diffusional trapping as this would imply the time between events to have a larger dispersion, to change as the NP size decreased during dissolution and to be independent of the collector electrode material. Fig. 11 shows a schematic representation of the periodic stripping process recorded using GC (11a) and Au (11b) as collector electrode. In the first case, the NP enters the tunnelling region and electro-oxidation of Ag atoms from the part of the NP in closer contact with the collector electrode occurs. As a result, the interaction between the NP (which could be pushed away by electrochemical propulsion^14^), due to a non-uniform electrochemical flux, and the substrate is momentarily broken. This absence of current lasts for just 0.5 ms. The process starts again and is repeated until the NP is completely consumed. For the Au collector electrode, the periodic stripping mechanism appears more complex, comprising two consecutive stripping events that occur within 25 ms that are followed by 50 ms without electrochemical current before repetition of the *I*–*t* motif.

The observation of a distinctly different periodic pattern on each of the two collector electrodes reflects some surface-specific features that can also be inferred from the analysis of the more prevalent irregular peak bundles (9c.iii) that we have discussed. These latter events occur on GC with a higher frequency and shorter duration than on Au (see Fig. 6c and 7). It is clear that the collector electrode material has a great influence on the stripping mechanism of impacting Ag NPs, as well as on the near-wall dynamics during partial stripping processes, and the investigation of these periodic patterns could be worthwhile in the future as a means of deepening understanding of NP–substrate interactions.

## 4 Conclusions

The detection of electrochemical reactions that occur upon impact of single NPs with an electrode surface – a growing field that is termed ‘single NP impact electrochemistry’ – requires that small signals can be measured quickly, necessitating low background currents (pA) and fast current amplifiers. Otherwise, the *I*–*t* signal becomes highly distorted, making it extremely difficult to interpret the underlying phenomena. These requirements have been met for the comprehensive time-resolved study presented herein on the electrochemical dissolution of single Ag NPs of different sizes by means of NP impacts in an SECCM configuration, using GC and Au as collector electrodes.

In contrast to previous work, it has been found that NP stripping leads to a wide range of very different and distinctive current transients (*I*–*t* morphologies), even during impacts of NPs of the same nominal size with the same substrate. Whereas most of the NPs of diameter, *d* = 10 nm dissolve electrochemically in single events (on the timescale of the measurement technique) that lasts a few milliseconds, this is not true of NPs with larger diameters. In this case, between 60% and 85% of the total consumed charge occurs in event bundles or saw-tooth current profiles that may span from tens of milliseconds up to several seconds for a single NP.

A quantitative analysis of the *I*–*t* features, consumed charge, event duration and impact frequency leads to a major conclusion: Ag NPs undergo consecutive partial stripping events in which a fraction of the NP is electrochemically oxidized, followed by the NP drifting away and then back to the tunnelling region for further (partial) dissolution. Whereas for NPs of *d* = 20 nm and 40 nm, the whole NP tends to be electrochemically dissolved after several consecutive (discrete) stripping events, the analysis of the associated *I*–*t* transients as individual events leads to the wrong estimation of the NP size distribution. Furthermore, for particles of *d* = 60 nm or 100 nm, most of the NPs are released from the near-wall region back into the bulk solution after incomplete stripping. Hence, the analysis of the charge consumed by single events (‘impact coulometry’) is not appropriate as a general method to determine the size of colloidal NPs.

On the other hand, we emphasize that a proper analysis of *I*–*t* transients provides very valuable information on the physicochemical interactions between NPs and polarized surfaces. In this work, we have shown that the relatively stronger interaction between Ag NPs and Au results in larger charges consumed per event, and longer residence time of the Ag NPs in the vicinity of the surface. In addition, an interesting, but rare, observation has been the detection of periodic *I*–*t* patterns with frequencies as high as 1 kHz, recorded for most of the NP sizes studied on both GC and Au. Whereas understanding the physical origin of such patterns requires further study, they serve to highlight the complexity of electrochemical dissolution phenomena *via* single NP impacts and the need for sub-ms time resolution (or, in the future, better) to properly study such processes.

## References

[cit1] Burda C., Chen X., Narayanan R., El-Sayed M. A. (2005). Chem. Rev..

[cit2] Tao A. R., Habas S., Yang P. (2008). Small.

[cit3] Aricò A., Bruce P., Scrosati B. (2005). Nat. Mater..

[cit4] Kleijn S. E. F., Lai S. C. S., Koper M. T. M., Unwin P. R. (2014). Angew. Chem., Int. Ed..

[cit5] Xiao X., Bard A. J. (2007). J. Am. Chem. Soc..

[cit6] Heinze J. (1993). Angew. Chem., Int. Ed. Engl..

[cit7] Kwon S. J., Bard A. J. (2012). J. Am. Chem. Soc..

[cit8] Alligrant T. M., Nettleton E. G., Crooks R. M. (2013). Lab Chip.

[cit9] Alligrant T. M., Dasari R., Stevenson K. J., Crooks R. M. (2015). Langmuir.

[cit10] Bard A. J., Zhou H., Kwon S. J. (2010). Isr. J. Chem..

[cit11] Robinson D. A., Yoo J. J., Castañeda A. D., Gu B., Dasari R., Crooks R. M., Stevenson K. J. (2015). ACS Nano.

[cit12] Xiao X., Fan F.-R. F., Zhou J., Bard A. J. (2008). J. Am. Chem. Soc..

[cit13] Dick J. E., Bard A. J. (2016). J. Am. Chem. Soc..

[cit14] Kang M., Perry D., Kim Y. R., Colburn A. W., Lazenby R. A., Unwin P. R. (2015). J. Am. Chem. Soc..

[cit15] Kleijn S. E. F., Lai S. C. S., Miller T. S., Yanson A. I., Koper M. T. M., Unwin P. R. (2012). J. Am. Chem. Soc..

[cit16] Zhou Y.-G., V Rees N., Compton R. G. (2011). Angew. Chem., Int. Ed..

[cit17] Lees J. C., Ellison J., Batchelor-Mcauley C., Tschulik K., Damm C., Omanovic̈ D., Compton R. G. (2013). ChemPhysChem.

[cit18] Bartlett T. R., Sokolov S. V., Compton R. G. (2015). ChemistryOpen.

[cit19] Toh H. S., Jurkschat K., Compton R. G. (2015). Chem.–Eur. J..

[cit20] Batchelor-McAuley C., Ellison J., Tschulik K., Hurst P. L., Boldt R., Compton R. G. (2015). Analyst.

[cit21] Sokolov S. V., Tschulik K., Batchelor-McAuley C., Jurkschat K., Compton R. G. (2015). Anal. Chem..

[cit22] Haddou B., Rees N. V., Compton R. G. (2012). Phys. Chem. Chem. Phys..

[cit23] Giovanni M., Ambrosi A., Sofer Z., Pumera M. (2015). Electrochem. Commun..

[cit24] Stuart E. J. E., Tschulik K., Batchelor-Mcauley C., Compton R. G. (2014). ACS Nano.

[cit25] Qiu D., Wang S., Zheng Y., Deng Z. (2013). Nanotechnology.

[cit26] Brasiliense V., Berto P., Combellas C., Kuszelewicz R., Tessier G., Kanoufi F. (2016). Faraday Discuss..

[cit27] Brasiliense V., Patel A. N., Martinez-Marrades A., Shi J., Chen Y., Combellas C., Tessier G., Kanoufi F. (2016). J. Am. Chem. Soc..

[cit28] Patel A. N., Martinez-Marrades A., Brasiliense V., Koshelev D., Besbes M., Kuszelewicz R., Combellas C., Tessier G., Kanoufi F. (2015). Nano Lett..

[cit29] Krause K. J., Adly N., Yakushenko A., Schnitker J., Mayer D., Offenhäusser A., Wolfrum B. (2016). Anal. Chem..

[cit30] Brasiliense V., Berto P., Combellas C., Tessier G. (2016). Acc. Chem. Res..

[cit31] Saw E. N., Grasmik V., Rurainsky C., Epple M., Tschulik K. (2016). Faraday Discuss..

[cit32] Sepunaru L., Sokolov S. V., Holter J., Young N. P., Compton R. G. (2016). Angew. Chem., Int. Ed..

[cit33] Kätelhön E., Tanner E. E. L., Batchelor-McAuley C., Compton R. G. (2016). Electrochim. Acta.

[cit34] Krause K. J., Yakushenko A., Wolfrum B. (2015). Anal. Chem..

[cit35] Kätelhön E., Compton R. G. (2014). Chem. Sci..

[cit36] Kang M., Momotenko D., Page A., Perry D., Unwin P. R. (2016). Langmuir.

[cit37] Chen C. H., Ravenhill E. R., Momotenko D., Kim Y. R., Lai S. C. S., Unwin P. R. (2015). Langmuir.

[cit38] Zhao L.-J., Qian R.-C., Ma W., Tian H., Long Y.-T. (2016). Anal. Chem..

[cit39] Kim Y. R., Lai S. C. S., McKelvey K., Zhang G., Perry D., Miller T. S., Unwin P. R. (2015). J. Phys. Chem. C.

[cit40] Lai S. C. S., Lazenby R. a., Kirkman P. M., Unwin P. R. (2015). Chem. Sci..

[cit41] Byers J. C., Paulose Nadappuram B., Perry D., McKelvey K., Colburn A. W., Unwin P. R. (2015). Anal. Chem..

[cit42] Bentley C. L., Kang M., Unwin P. R. (2016). J. Am. Chem. Soc..

[cit43] Edwards M. A., Williams C. G., Whitworth A. L., Unwin P. R. (2009). Anal. Chem..

[cit44] Morgan D. M., Weber S. G. (1984). Anal. Chem..

[cit45] Nomura S., Nozaki K., Okazaki S. (1991). Anal. Chem..

[cit46] Lee H. M., Ge M., Sahu B. R., Tarakeshwar P., Kim K. S. (2003). J. Phys. Chem. B.

[cit47] Khomyakov P. A., Giovannetti G., Rusu P. C., Brocks G., Van Den Brink J., Kelly P. J. (2009). Phys. Rev. B: Condens. Matter Mater. Phys..

[cit48] Kwon S. J., Fan F. R. F., Bard A. J. (2010). J. Am. Chem. Soc..

[cit49] Kwon S. J., Zhou H., Fan F.-R. F., Vorobyev V., Zhang B., Bard A. J. (2011). Phys. Chem. Chem. Phys..

[cit50] Bevan M. A., Prieve D. C. (2000). J. Chem. Phys..

[cit51] Ustarroz J., Ke X., Hubin A., Bals S., Terryn H. (2012). J. Phys. Chem. C.

[cit52] Ustarroz J., Hammons J. A., Altantzis T., Hubin A., Bals S., Terryn H. (2013). J. Am. Chem. Soc..

[cit53] Chen C.-C., Zhou Y., Baker L. A. (2011). ACS Nano.

[cit54] Chazalviel J. N., Allongue P. (2011). J. Am. Chem. Soc..

