# Supplementary material for: Impact and oxidation of single silver nanoparticles at electrode surfaces: one shot versus multiple events
Source: Chem Sci. 2016 Dec 12;8(3):1841–53. doi: 10.1039/c6sc04483b (PMC5424807; doi:10.1039/c6sc04483b)
Supplement: Supplementary file 1 [file SC-008-C6SC04483B-s001.pdf]

Electronic Supplementary Material (ESI) for Chemical Science.
This journal is © The Royal Society of Chemistry 2016

Supporting Information

Impact and Oxidation of Single Silver Nanoparticles at Electrode Surfaces: One Shot versus Multiple Events

Jon Ustarroz+, Minkyung Kang+, Erin Bullions and Patrick R. Unwin**

Department of Chemistry, University of Warwick, Coventry, CV4 7AL, United Kingdom

Research Group Electrochemical and Surface Engineering (SURF), Vrije Universiteit Brussel, Pleinlaan 2, 1050 Brussels, BelgiumAddress

+ *Contributed equally to this work*

*p.r.unwin@warwick.ac.uk

*jon.ustarroz@vub.ac.be

CONTENTS

Section S1 Conditions for previous studies of Ag NP electro-oxidation S3

Section S2 Ag NP characterization: TEM and DLS S5

Section S3 Event Duration Histograms (Log-Log) S11

Section S4 Max Current Histograms (Log-Log) S12

Section S5 Citrate effect on Ag electrodissolution S13

References S14

S1 Conditions for previous studies of Ag NP electro-oxidation

Table S1 Summary of Conditions for previous studies of Ag NP electro-oxidation.

Reference number[a]	Electrodes[b] (μm)	NP Size[c] (nm)	Capping agent[d] and electrolyte	E_{app}[e] vs reference electrode[f]
Ref [16]	GC-UME<br>(d=N/A)	20-50	Citrate<br>10 mM Sodium dihydrogen citrate and 90 mM KCl	*I-t* (not specified)<br>Varied E (50-500 mV) for *I-V* curve vs Ag/AgCl
Ref [17]	CF-UME (d=10)	17-48	Citrate<br>100 mM trisodium citrate	0.6 V vs. Ag wire<br>or<br>0.3V vs MSE
Ref [18]	GC-UME<br>(d=11.3)	100	Citrate<br>20 mM KCl	0.6V vs SCE
Ref [19]	CF-UME (d=10)	30	Different capping agents including citrate<br>20 mM $NaNO_3$	0.6 V vs MSE
Ref [20]	CF-UME (d=7)	12	Citrate<br>20 mM trisodium citrate	0.6 V vs. MSE
Ref [21]	CF-UME (d=7)-RAM	100	Citrate<br>0 -2.5 M KCl	0.6 V vs SCE
Ref [27]	Au square UME<br>(50 X 50 □m^2)	100	Citrate<br>50 mM KNO_3	0.6-0.9 V<br>vs Ag/AgCl QRE
Ref [28]	Au coated glass electrode<br>(7 mm^2)	60 and 100	Capping agent –not specified<br>30-50 mM KSCN and KNO_3	Various potentials<br>vs Ag/AgCl QRE
Ref [29]	Pt-UME (d=8-20)	20	Citrate<br>KCl (concentration not specified)	0.4 V vs Ag/AgCl

Ref [31]	Array of Pt(d=8)-UME	10	Citrate<br>120 mM KCl	0.4 vs Ag/AgCl

[a]Reference number is originated from main text. [b]UME, ultramicroelectrode; RAM, random assembly microelectrode. [c]All NP sizes are presented as diameter. [d]Solutions contain citrate, but the concentration is not always easily determined from the AgNP colloidal solutions being used. [e]E_{app}, applied potential at the collector electrode. [f]Ag/AgCl, commercial Ag/AgCl reference electrode; SCE, saturated calomel electrode; MSE, mercury sulfate electrode; Ag/AgCl QRE, Ag/AgCl quasi reference electrode.

S2. Ag NP characterization: TEM and DLS

Silver nanoparticles (AgNPs) with nominal diameter of 10, 20, 40, 60 and 100 nm (abbreviated as Ag10NPs, Ag20NPs, Ag40NPs, Ag60NPs and Ag100NPs, respectively) were characterized by dynamic light scattering (DLS) and transmission electron microscopy (TEM). Representative TEM images are shown in Figure S1. The average diameters obtained by both TEM and DLS are shown in Table S1. DLS measurements after addition of 25 mM $NaNO_3$ also performed to rule out NP agglomerations induced by the electrolyte (Table S1).

The concentrations of Ag NPs were estimated based on weight per volume concentration (provided by the supplier) considering molecular weight (107.86 g mol^{-1}) and bulk density (10.49 g cm^{-3}) (Table S2). The bulk diffusion coefficient of NPs (D_{NP}) was also calculated by the Stokes-Einstein equation:

$$D_{\mathrm{NP}} = \frac{k_{\mathrm{B}}T}{6\pi\eta r_{\mathrm{NP}}} \quad (1)$$

where k_B is the Boltzmann constant (1.381×10^{-23} J K^{-1}), T is the temperature (298 K), η is the dynamic viscosity of the solution (8.90×10^{-4} Pa s for dilute aqueous solution), and r_{NP} is the NP radius from the TEM results in Table S1 (Table S2).

For a diffusion-controlled movement of the NPs towards the collector surface, the estimated impact frequency (f_{NP}) can be calculated by the following equation.[1]

$$f_{\mathrm{NP}} = 0.52 D_{\mathrm{NP}} C_{\mathrm{NP}} N_{\mathrm{A}} r_{\mathrm{disc}} \quad (2)$$

where D_{NP} is the diffusion coefficient of NPs of the given sizes (Table S2), C_{NP} is the concentration of NPs (Table S2), N_A is the Avogadro constant (6.022×10^{23} mol^{-1}), and r_{disc} is the radius of the electrode (3.1 μm herein considering the expansion of meniscus after land on the surface (FigureS2)). The diffusion flux in the SECCM setup herein was 13 % of a disc UME of the same size, deduced by measuring the steady-state current in 2 mM

$Ru(NH_3)_6^{3+}$ including 0.1 M KNO_3 (Figure 2S).[2] This resulted in using 0.52 in eq (2) instead of a factor of 4. The f_{NP} results of each type of AgNP are presented in Table S2.

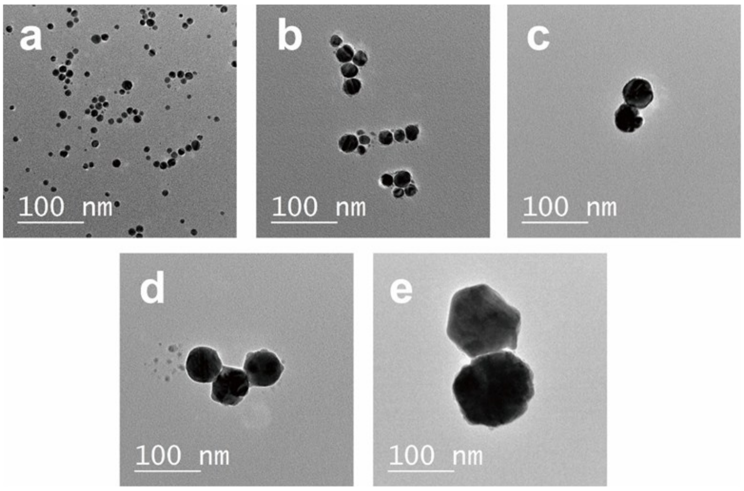

Figure S1 Representative TEM images of Ag NPs with nominal diameters of (a) 10 nm, (b) 20 nm, (c) 40 nm, (d) 60 nm and (e) 100 nm (abbreviated as Ag10NPs, Ag20NPs, Ag40NPs, Ag60NPs and Ag100NPs, respectively).

Table S2 Average diameters of Ag NPs as determined from TEM and DLS.

	**TEM**	**DLS (w/o 25 mM $NaNO_3$)**	**DLS (w/ 25 mM $NaNO_3$)**
**Ag10NPs**	9.4 ± 0.2	13.2 ± 1.8	12.1 ± 1.0
**Ag20NPs**	19.4 ± 1.6	22.5 ± 1.6	21.2 ± 1.0
**Ag40NPs**	39 ± 0.6	40.3 ± 1.0	39.0 ± 1.1
**Ag60NPs**	58.2 ± 1	72.7 ± 2.2	72.8 ± 6.0
**Ag100NPs**	93.8 ± 1.4	91.7 ± 5.9	90.5 ± 2.7

The size characterization from TEM and DLS shows good agreement. DLS provides slightly larger values than TEM because hydrodynamic diameters are calculated from DLS measurements. The DLS results after addition of 25 mM $NaNO_3$ showed that NP agglomerations induced by the electrolyte were negligible for the timescale for the measurements (Table S1).

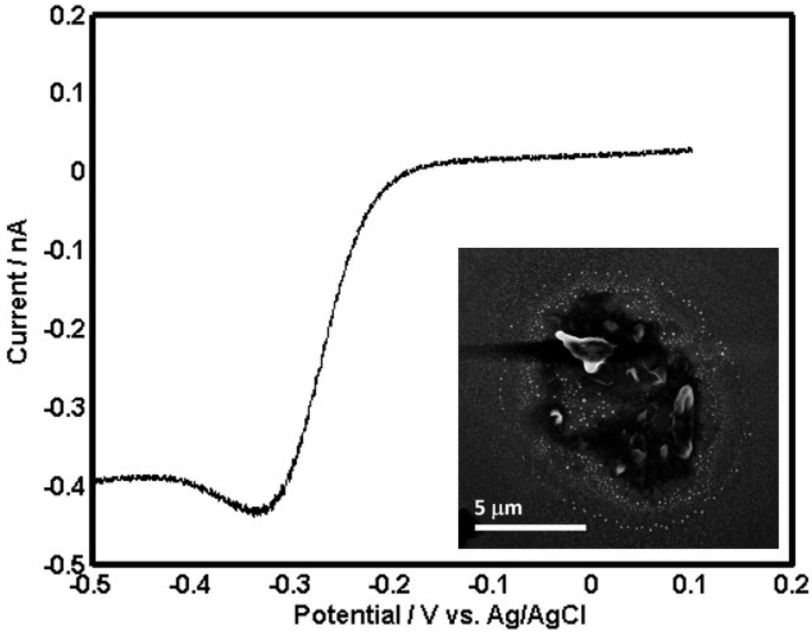

Figure S2 Linear sweep voltammogram (LSV) (100 mV s^{-1}) for the reduction of 2 mM $Ru(NH_3)_6^{3+}$ in 0.1 M KNO_3 solution by the meniscus contact on GC using a glass pipette (diameter of 8 μm) and SEM image of the footprint after the meniscus contact (inset). The diameter of the droplet was just 25 % larger than the pipette end diameter. Note that the LSV is not fully at steady-state due to the scan speed used and the fact that SECCM diffusion is from a conical segment rather than fully hemispherical.

Table S3 Estimated concentration, diffusion coefficient and impact frequency of distributions of Ag NPs with nominal diameters of 10, 20, 40, 60 and 100 nm.

	**Estimated concentration (M)**	D_{NP} **(cm^2 s^{-1})**	f_{NP} **(s^{-1})**
**Ag10NPs**	6.0 10^{-9}	5.2 10^{-7}	240
**Ag20NPs**	7.6 10^{-10}	2.5 10^{-7}	15
**Ag40NPs**	9.5 10^{-11}	1.3 10^{-7}	0.94
**Ag60NPs**	2.8 10^{-11}	8.4 10^{-8}	0.19
**Ag100NPs**	6.0 10^{-12}	5.2 10^{-8}	0.025

S3 Event Duration Histograms (Log-Log)

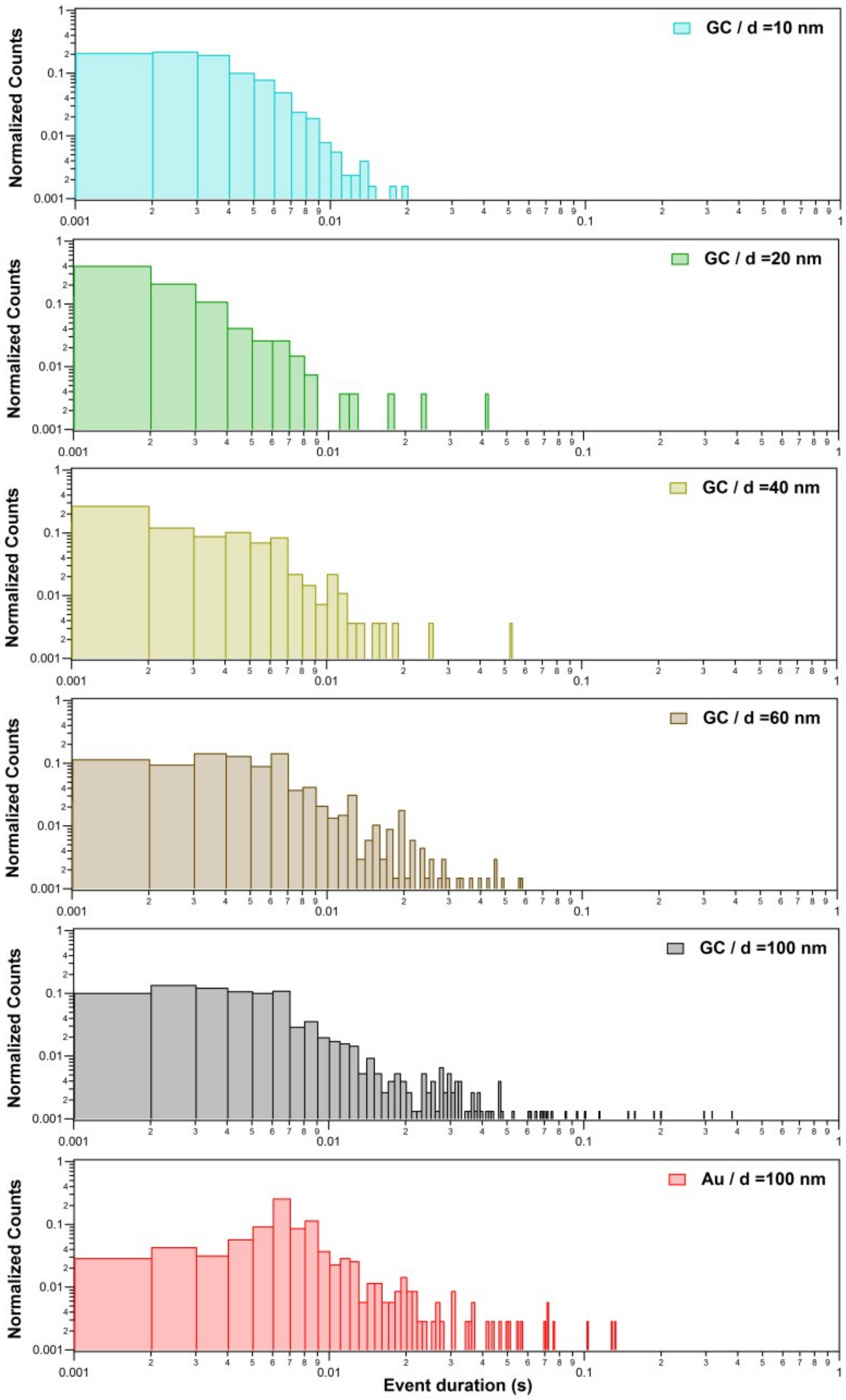

Figure S3 Log-log plot of the event duration histograms for single events recorded during the stripping of Ag NPs with nominal diameter of 10, 20, 40, 60 and 100 nm on GC and Au electrodes.

S4 Max Current Histograms (Log-Log)

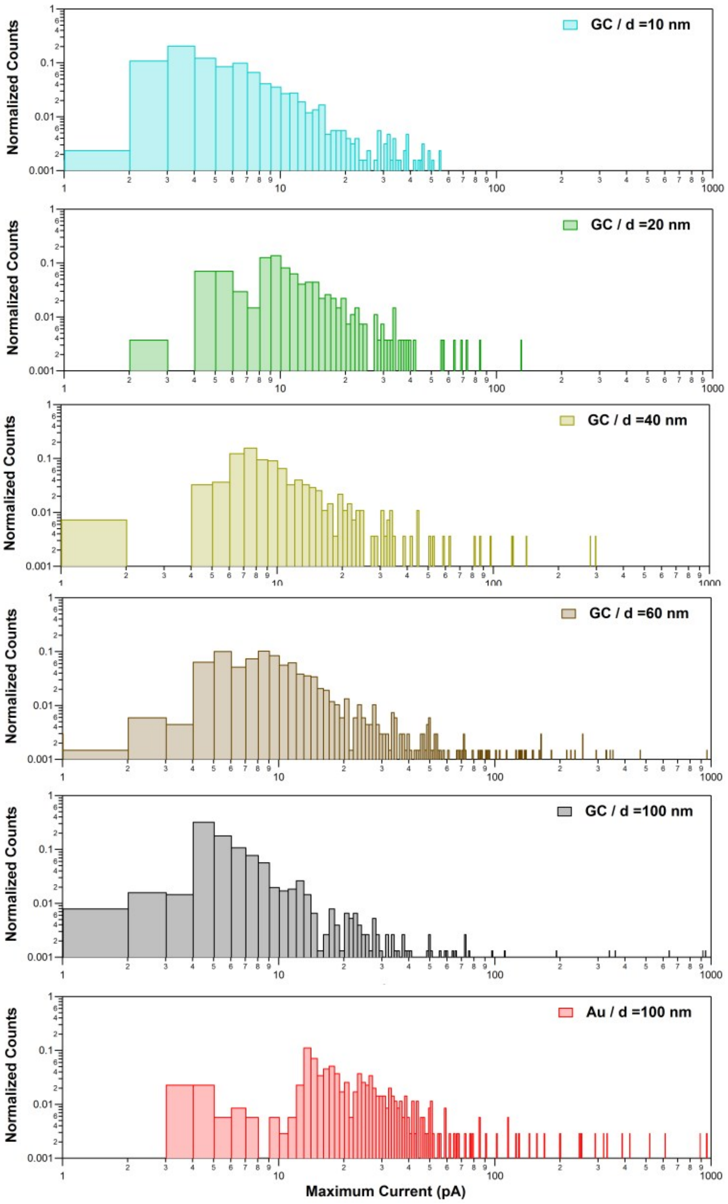

Figure S4 Log-log plot of the maximum current histograms for single events recorded during the stripping of Ag NPs with nominal diameter of 10, 20, 40, 60 and 100 nm on GC and Au electrodes.

Section S5 Citrate effect on Ag electrodissolution

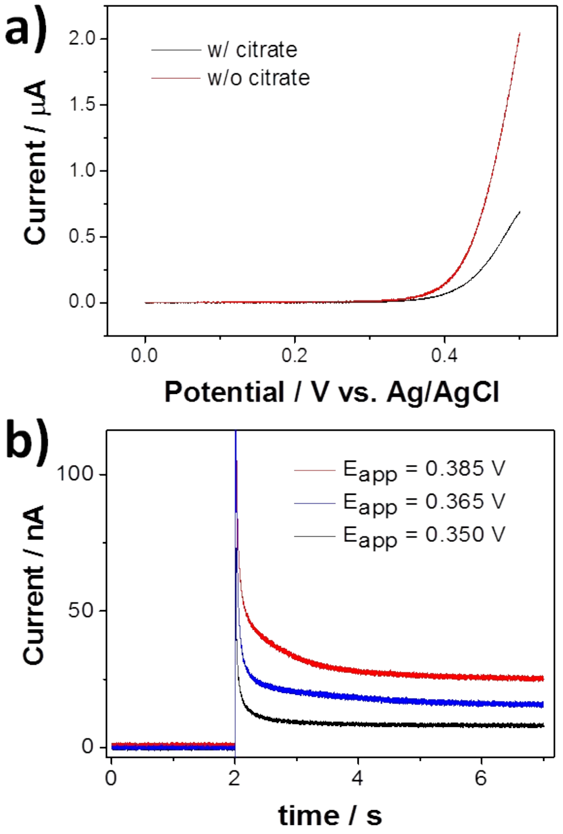

Figure S5 (a) LSV (50 mV s^{-1}) for Ag (UME of diameter = 125 μm) electrodissolution in presence (black) and absence (red) of 1 mM trisodium citrate in 25 mM $NaNO_3$. (b) Current-time curves at different applied potentials (E_{app} vs. Ag/AgCl) where the steady-state currents are corresponding to 0.6 (black), 1.3 (blue), and 1.9 (red) mM of Ag^+ concentrations on the Ag UME surface. Citrate inhibits Ag electrodissolution (probably by surface adsorption) but there is no evidence of surface passivation due to Ag_3Cit precipitation.

References

(1) Xiao, X.; Bard, A. J. *J. Am. Chem. Soc.* **2007**, *129*, 9610.

(2) Snowden, M. E.; Güell, A. G.; Lai, S. C. S.; McKelvey, K.; Ebejer, N.; O'Connell, M. A.; Colburn, A. W.; Unwin, P. R. *Anal. Chem.* **2012**, *84*, 2483.
